# Combined central retinal vein occlusion and cilioretinal artery occlusion as the initial presentation of frosted branch angiitis: a case report and literature review

**DOI:** 10.1186/s12348-023-00340-7

**Published:** 2023-05-25

**Authors:** Abdullah Albahlal, Waleed Khayyat, Sulaiman M. Alsulaiman

**Affiliations:** King Khaled Eye Specialist Hospital, Riyadh, Kingdom of Saudi Arabia

**Keywords:** Central retinal vein occlusion, Frosted branch angiitis, Cilioretinal artery occlusion, Uveitis, Behcet’s disease

## Abstract

**Purpose:**

To report a case of combined central retinal vein occlusion (CRVO) with cilioretinal artery occlusion (CLRAO) that heralded the development of frosted branch angiitis (FBA).

**Case report:**

A 25-year-old healthy male presented with sudden painless visual loss in his left eye with a visual acuity (VA) of 20/300. Fundus exam and fluorescein angiography showed signs of combined CRVO and CLRAO. Without treatment, his vision gradually improved until it reached 20/30 within four months. Five months after initial presentation, he returned with severe visual loss (20/400) in the same eye and a clinical picture of severe occlusive periphlebitis resembling a frosted branch angiitis pattern associated with severe macular edema. This was promptly and successfully treated with systemic steroids and immunosuppressive medications.

**Conclusion:**

CRVO in young population can have an unusual course and one should carefully rule out underlying uveitic etiologies in each visit. Clinical suspicion and close follow‑up are required for early detection and timely management of FBA.

## Introduction

Frosted branch angiitis (FBA) is a rare form of retinal vasculitis characterized by a fulminant retinal perivascular sheathing involving the venules (and occasionally both arterioles and venules), with varying degrees of uveitis, macular edema (ME) and visual loss [1–3].

To the best of our knowledge, there are only about 11 reported cases of FBA associated with central retinal vein occlusion (CRVO). In all of these cases, the vascular occlusion developed either concurrent or subsequent to FBA. We report the first case in which a combined CRVO and cilioretinal artery occlusion (CLRAO) preceded the development of FBA.

## Case report

**Fig. 1:**
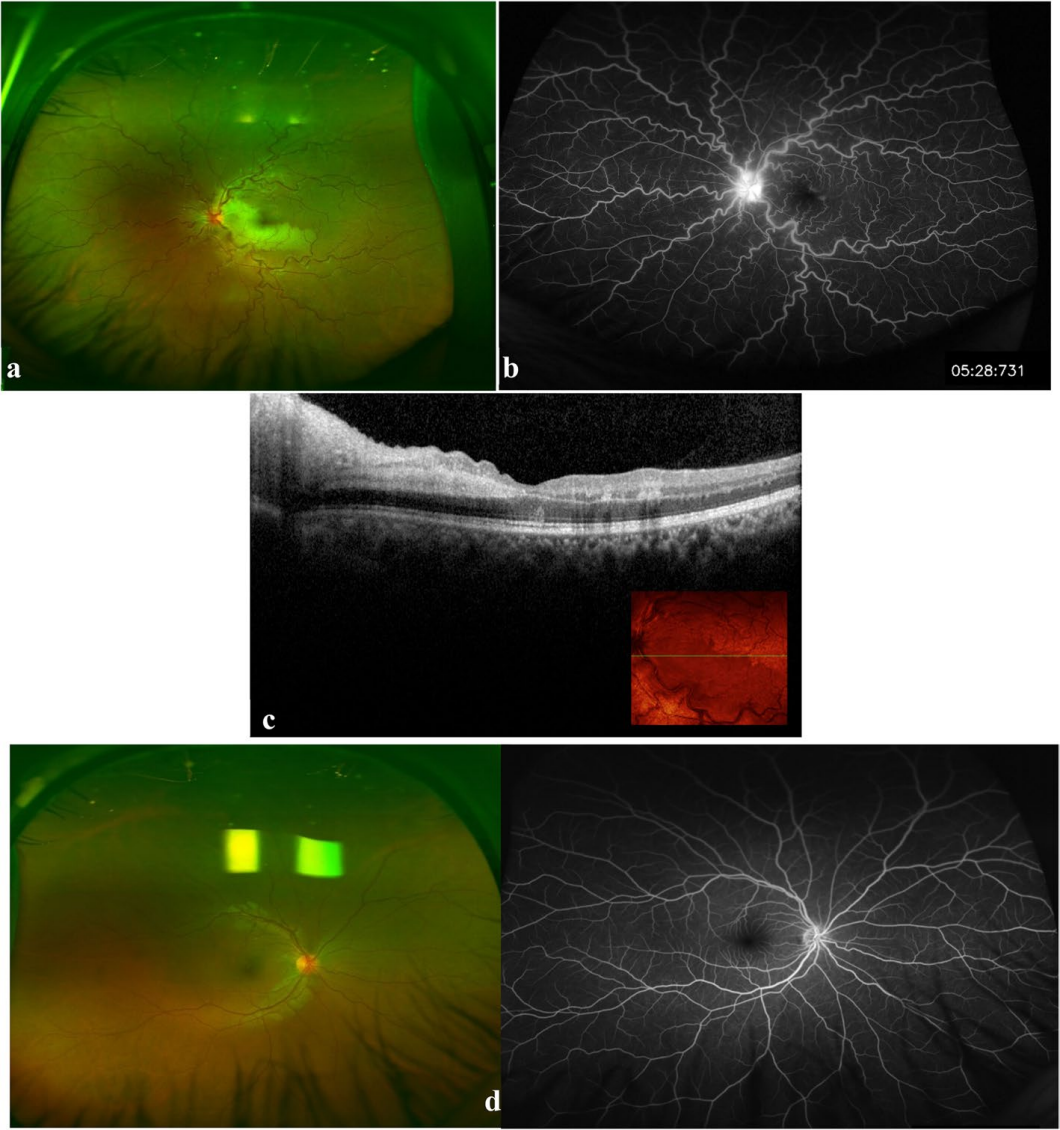
Imaging at initial presentation. **a** Color fundus photograph of the left eye showing dilated tortuous veins, swollen optic disc, and ischemic retinal whitening at the distribution of cilioretinal artery, Note: the two white areas above the superior retinal arcades represent a reflection artefact from the fundus camera. **b** A fluorescein angiogram showing disc leakage, with no abnormal vascular leakage or capillary non-perfusion (note: consecutive angiography frames revealed an obvious delay in the venous filling). **c** SD-OCT image showing inner retinal hyperreflectivity and thickening involving the nasal macula in the left eye. **d** Color fundus photo and fluorescin angiogram image of the normal right eye

**Fig. 2:**
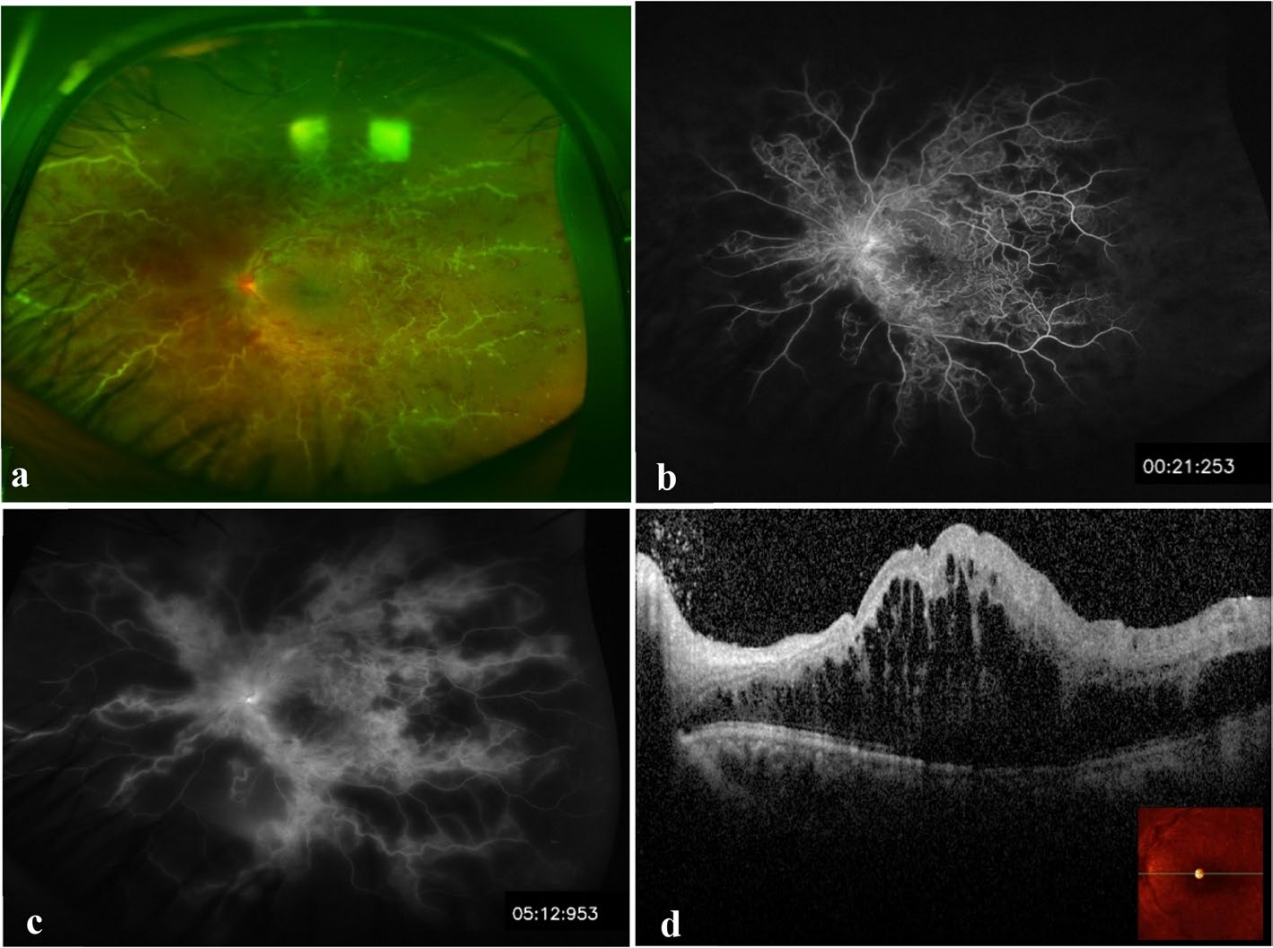
Imaging five months after the initial presentation. **a** The same eye shows increased disc edema with hyperemia, macular edema, and thick perivascular sheathing along the major vessels (predominantly venous), typical of frosted branch angiitis. **b** and **c** Early and late fluorescein angiograms revealed extensive retinal capillary non-perfusion and leakage from the optic nerve head and retinal veins. **d** SD-OCT shows severe macular edema

A 25-year-old healthy male presented to our clinic in 2018 with sudden painless loss of vision in the left eye (OS). The visual acuity (VA) was 20/300 at presentation. The patient had no history of recent systemic illness or vaccinations. Examination findings included normal anterior segment, clear vitreous, optic disc hyperemia, dilated and tortuous retinal veins along with retinal ischemic whitening of the cilioretinal artery territory (Fig. 1a). Fundus fluorescein angiography (FFA) revealed delayed venous filling and delayed emptying, and impaired filling of the cilioretinal artery, confirming the diagnosis of combined non-ischemic CRVO and CLRAO (Fig. 1b and c). Examination of the fellow eye was unremarkable (Fig. 1d), and remained unremarkable to the last follow-up. Systemic evaluation including blood pressure, fasting blood sugar, complete blood cell count (CBC), hemoglobin, C-reactive protein (CRP), erythrocyte sedimentation rate (ESR), urea, electrolytes, coagulation profile, cryoglobulins, antiphospholipid antibodies, factor V Leiden mutation, protein C and S levels, antithrombin III mutation, prothrombin mutation, homocysteine levels, serum protein electrophoresis, carotid ultrasound, and echocardiogram revealed no obvious abnormalities. His VA continued to improve, reaching 20/30 in four months. Five months following the initial presentation, he returned with a severe visual loss (20/400) in the left eye. He also had ciliary injection and an inflammatory anterior chamber reaction with a hypopyon of 0.2 mm height. Mild vitritis was also present accompanied by severe periphlebitis in a frosted branch pattern (Fig. 2a). A repeated FFA showed severe generalized retinal capillary non-perfusion sparing the macula (Fig. 2b and c). Spectral domain optical coherence tomography (SD-OCT) revealed a newly formed severe ME (Fig. 2d). Upon further detailed questioning, the patient recalled suffering from occasional oral ulcers. There was no history of genital ulcers, erythema nodosum of the skin, joint pain, neurological, nor gastrointestinal symptoms. The combined presence of oral and ocular lesions indicates a diagnosis of Behcet’s disease based on the international criteria for Behçet's disease [4].

The patient was admitted to the hospital for further investigation and management. Full medical examination was unremarkable; there were no clinical signs of lymphoma, leukemia, sarcoidosis, tuberculosis, multiple sclerosis, systemic lupus erythematosus, or other autoimmune diseases. Laboratory tests were carried out, including CBC, renal and liver function tests, angiotensin converting enzyme level, CRP, ESR, serum protein electrophoresis, HLA-B51, autoimmune markers (anticardiolipin antibodies, anti-neutrophil cytoplasmic antibodies, antinuclear antibodies, antimitochondrial antibodies, rheumatoid factor, anti-double-stranded DNA, anti-single stranded DNA, anti-Scl-70 antibodies, and anti-Jo-1 antibodies), and serological tests for syphilis, viral hepatitis and HIV. ESR was high 34 mm/h (baseline ESR = 7 mm/h). Results of all other tests were within normal limits or negative. Chest computed tomography scan and doppler ultrasonography of carotid arteries were also normal.

**Fig. 3:**
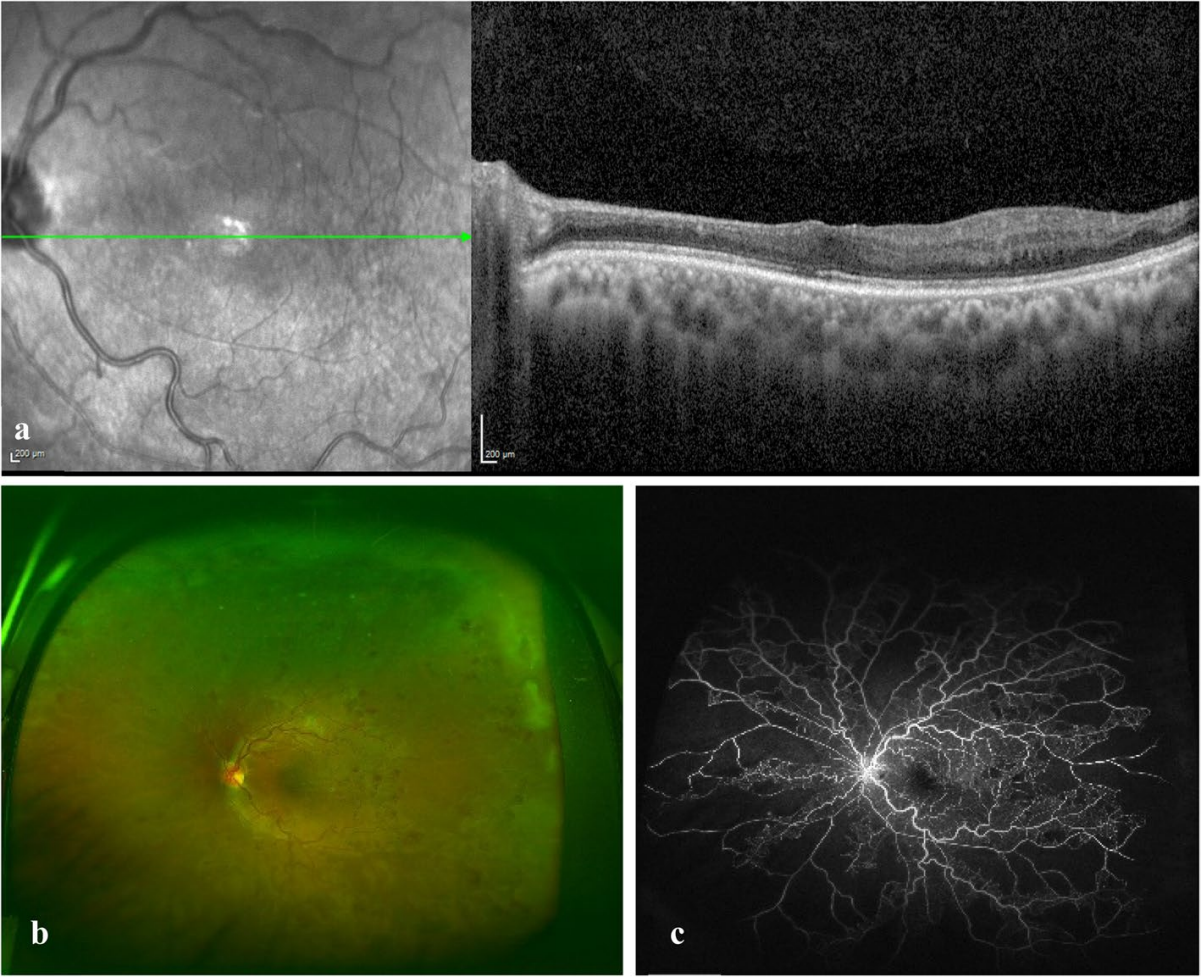
Imaging following treatment. **a** SD-OCT showed resolved macular edema following serial anti-VEGF injections and inner retinal atrophy from previous cilioretinal artery occlusion. At 12 months following immunosuppressive therapy, **b** fundus photo shows resolution of inflammatory signs, residual venous tortuosity and intraretinal hemorrhages and (c) late frame of fluorescein angiogram shows resolution of leakage from the optic nerve head and retinal veins and persistence of the retinal capillary non-perfusion

Two days later, following a negative tuberculin skin test, a pulse therapy of intravenous methylprednisolone (1 g/day for 3 days) was initiated followed by a tapering regimen of oral prednisolone (1 mg/kg) and immunosuppressive therapy (Azathioprine 1 mg/kg, increased later to 2 mg/kg). One week later, there was a marked resolution of the perivascular sheathing and retinal hemorrhages. Two months later, all signs of inflammation had resolved with a slight improvement in ME. Treatment for ME was started with monthly intravitreal bevacizumab (1.25 mg/0.05 ml), which resulted in a good response after completing the loading regimen (three injections). Five months later, the ME resurged after a period of non-compliance to maintenance immunosuppressive medications. The ME eventually resolved after additional two injections of bevacizumab followed by two injections of aflibercept (Fig. 3a) with a resultant VA of 20/25. Twelve months from commencing immunosuppressive therapy, there were no signs of inflammation (Fig. 3b and c). Later, sector laser photocoagulation was applied to the ischemic retina when small tufts of retinal neovascularization were noted.

## Discussion

Kleiner classified FBA according to the etiology into three groups [1]. The first group involves patients with lymphoproliferative disorders who have malignant infiltration of retinal vascular tree. The second group involves patients with autoimmune diseases and infections with FBA representing an ocular manifestation. The third, more common, group is termed (acute idiopathic FBA) and occurs in otherwise healthy individuals [2, 5]. Our patient may fit into the second group of FBA given the history of oral ulcers and the presence of mobile hypopyon. FBA in the setting of Behcet’s disease has been reported previously [6–12].

Eleven previous cases of retinal periphlebitis resembling FBA associated with CRVO have been documented (Table 1) [13–19]. In all of the cases, the CRVO had either accompanied FBA at presentation or occurred later at an interval between 2 to 16 weeks. Whereas in our case, CRVO preceded the onset of periphlebitis by about five months. This might be explained by the presence of an underlying systemic vasculitis that has a prothrombotic tendency (i.e., Behcet’s disease) [20].

**Table 1:** Previously reported cases of frosted branch angiitis associated with central retinal vein occlusion

Author	Age	Involved eye: Duration to CRVO	Associated Systemic Abnormality	Treatment	Complications	Final BCVA
Foss et al. 1992 [13]	23/M	LE: 4wks	None	Systemic CS, PRP	NVG	LP
	54/M	RE: simultaneous	None	PRP	CME	CF
Seo et al. 1998 [14]	27/M	LE: 4 months	None	Systemic CS, PRP	NVG, VH	HM
Kaburaki et al 2001 [15]	36/F	RE: 3wks	None	Systemic CS, PRP	NVG, VH	LP
	23/F	LE: 2wks	High RF	Systemic CS, PRP	NVG	LP
Abu El-Asrar et al. 2003 [16]	28/M	RE: 2wks LE: -10 days	Carotid artery stenosis Antiphospholipid antibodies	Systemic CS, azathioprine, Plasmapheresis, PRP	OD: phthisis bulbi OS: TRD OU: NVG	NLP OU
	47/M	LE: simultaneous	Antiphospholipid antibodies High Homocysteine	Systemic CS, PRP	Rubeosis irides	N/A
Satoh et al 2010 [17]	39/M	LE: -4 days	Familial Mediterranean Fever	Systemic CS, Antiviral, Antibacterial	N/A	20/20
Greifner et al 2016 [18]	37/M	LE: 3 months	None	Systemic CS, Anti-VEGF	CME	20/30
	45/F	RE: 4 months	None	Systemic CS, Anti-VEGF	CME	20/50
Kumawat et al. 2017 [19]	28/M	LE: -3 days	None	Systemic CS	CME	20/20

*Abbreviations*: *CRVO* Central retinal vein occlusion, *BCVA* Best-corrected visual acuity, *CS* Corticosteroids, *LP* Light perception, *NLP* No light perception, *CF* Counting fingers, *HM* Hand motion, *RE* Right eye, *LE* Left eye, *VH* Vitreous hemorrhage, *NVG* Neovascular glaucoma, *TRD* Tractional retinal detachment, *CME* Cystoid macular edema, *PRP* Pan-retinal photocoagulation, *VEGF* Vascular endothelial growth factor

Anti-tumor necrosis factor therapy is becoming the first-line treatment in Behcet’s disease and it was considered at some point in the management of this patient [21, 22]. However, given the stabilization of the condition on conventional immunosuppressive therapy for several years, and the recovery of good visual acuity in the affected eye, we elected to continue the current management.

Retinal ischemia and proliferative retinopathy represent a clinical challenge when associated with fulminant ocular inflammation. Aggressive control of inflammation is an important prerequisite for involution of retinal neovascularization. Classically, laser photocoagulation targeting the ischemic retina has been considered as the treatment of choice [23–25]. Some authors advocate early laser treatment before the development of neovascularization [26]. However, laser may need to be used judiciously in an eye with fulminant inflammation as it was linked to an upregulation of cytokines and other inflammatory mediators. This pro-inflammatory effect is especially well-demonstrated in the studies of progression of diabetic macular edema following panretinal photocoagulation [27–30]. Moreover, other investigators have documented an increase in the vitreous humor levels of vascular endothelial growth factor (VEGF) and several proinflammatory cytokines in mice and rabbits following laser burns [30, 31]. This pro-inflammatory effect of retinal laser is less pronounced in the modern short-pulse laser technology [30]. In our patient, we used anti-VEGF therapy to address ME and suppress retinal neovascularization. Once we noted the development retinal neovascularization, we immediately applied laser photocoagulation to the ischemic retina.

This case presents a unique challenge as it initially presented with CRVO/CLRAO without frank signs of inflammation on clinical examination or FFA. An inflammatory etiology of CRVO in young population should be carefully investigated. Clinical suspicion and close follow‑up are required for early detection and timely management of FBA.

## Data Availability

Not applicable.

## Abbreviations

CRVO: Central retinal vein occlusion
CLRAO: Cilioretinal artery occlusion
OS: Left eye
VA: Visual acuity
FBA: Frosted branch angiitis
ME: Macular edema
FFA: Fundus fluorescein angiography
CBC: Complete blood cell count
CRP: C-reactive protein
ESR: Erythrocyte sedimentation rate
SD-OCT: Spectral domain optical coherence tomography
VEGF: Vascular endothelial growth factor
